# Supplementary figures and images for: Analysis of fungal dynamic changes in the natural fermentation broth of ‘Hongyang’ kiwifruit
Source: PeerJ. 2022 Apr 19;10:e13286. doi: 10.7717/peerj.13286 (PMC9029446; doi:10.7717/peerj.13286)

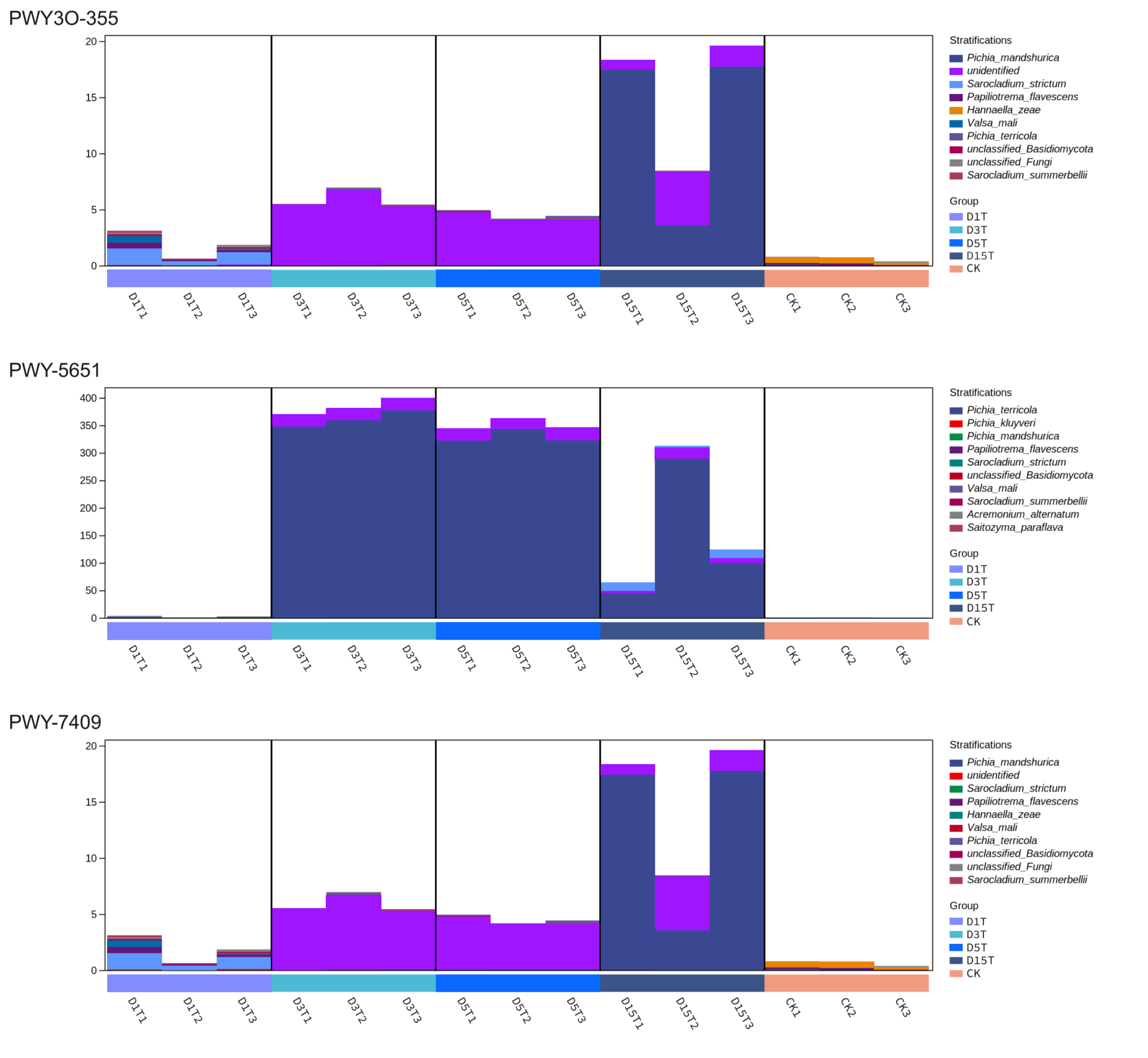

Supplement: Supplemental Information 2 — Among the three pathways, the dominant species of D3T, D5T, and D15T were Pichia, while D1T was not. These three pathways were inferred to be regulated by Pichia. [file peerj-10-13286-s002.png]

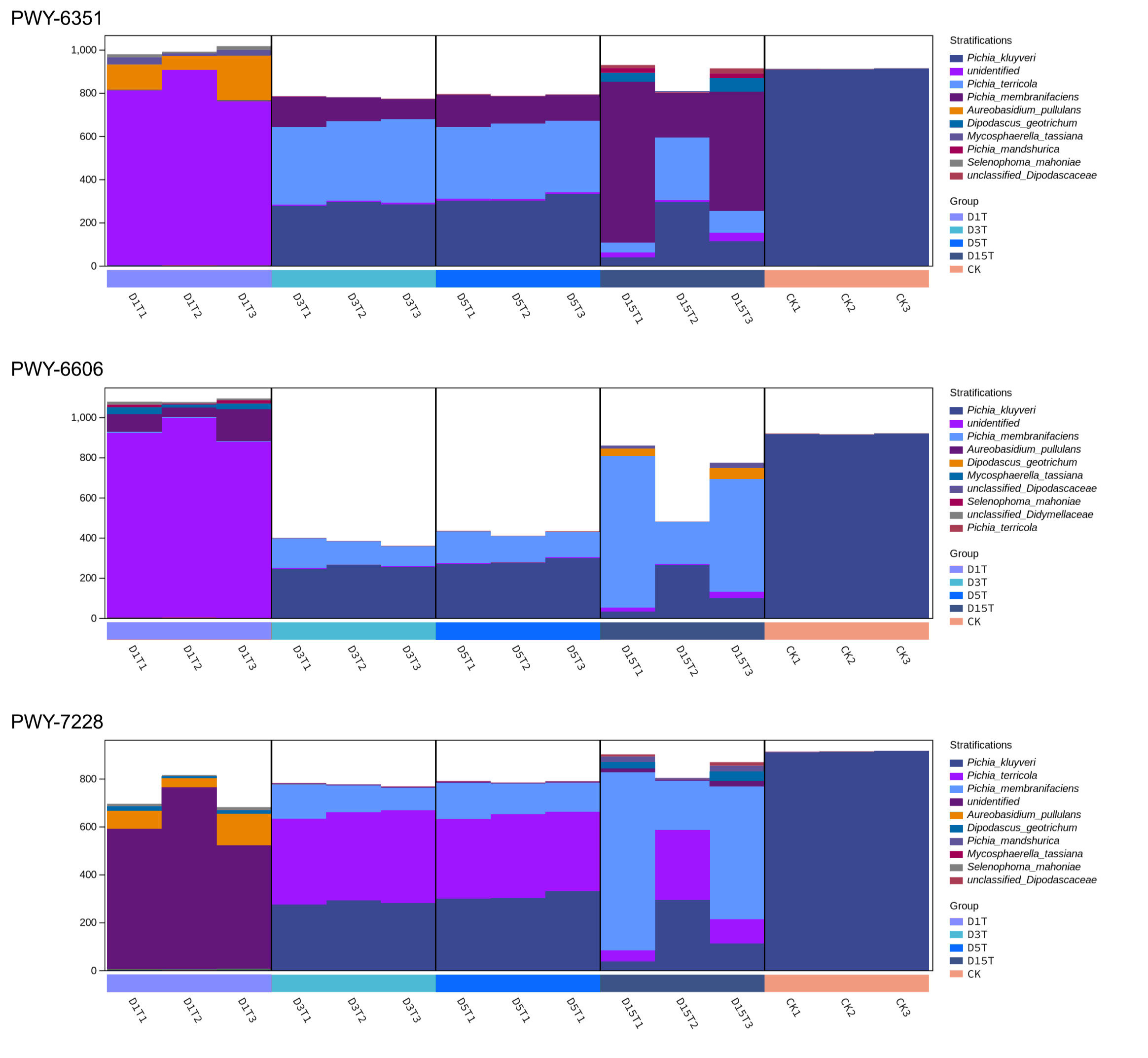

Supplement: Supplemental Information 3 — The dominant species of D3T, D5T, and D15T in the three Pathways were Pichia, while those of D1t were not. These three pathways were inferred to be regulated by Pichia. [file peerj-10-13286-s003.png]

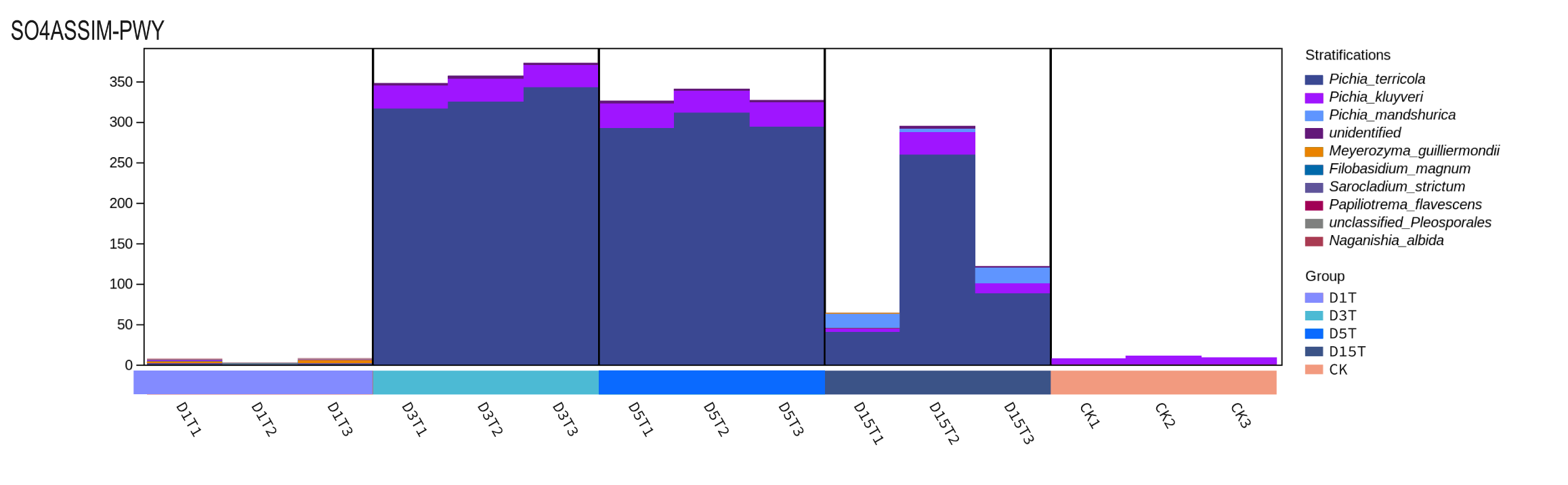

Supplement: Supplemental Information 4 — Pichia was found in D1T, D3T, D5T and D15T. Among them, the number of Pichia in D1T was much lower than that of CK, and the abundance of Pichia in D3, D5, and D15 was much higher than that in CK. Therefore, this pathway was regulated by Pichia. [file peerj-10-13286-s004.png]

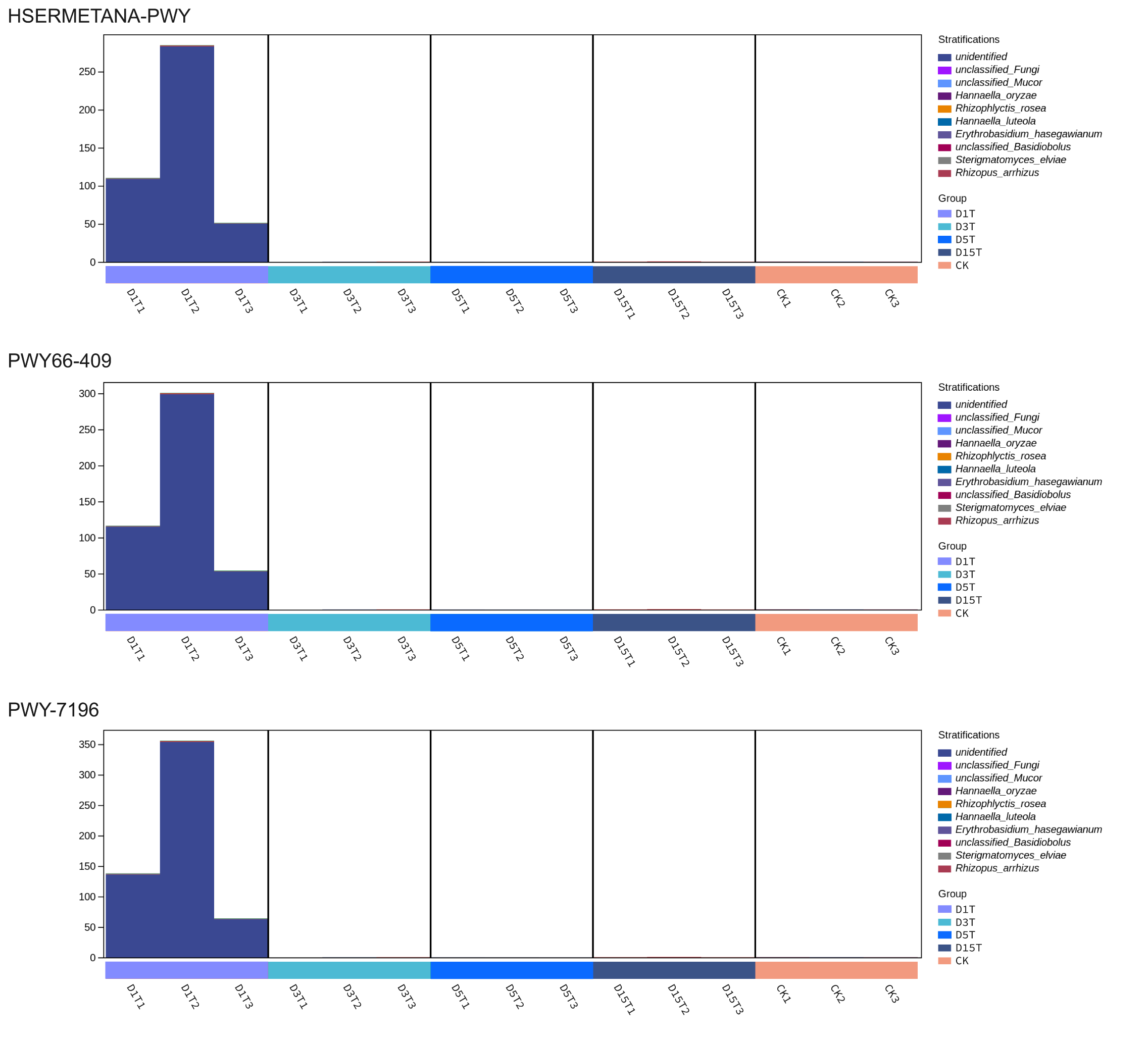

Supplement: Supplemental Information 5 — The dominant species of D1T, D3T, D5T, and Ck were all unidentified. With the progress of natural fermentation going on, the dominant species of unclassified had been eliminated or reduced and become unclassified Fungi in D15T. Among them, the abundance of unidentified in D1T was much greater than that of CK. Therefore, D1T was up-regulated compared with the control group. The abundances of unidentified in D3T and D5T were much less than CK, these pathways were down-regulated. There was no unidentified in D15T, and therefore these pathways were not found in D15T. [file peerj-10-13286-s005.png]

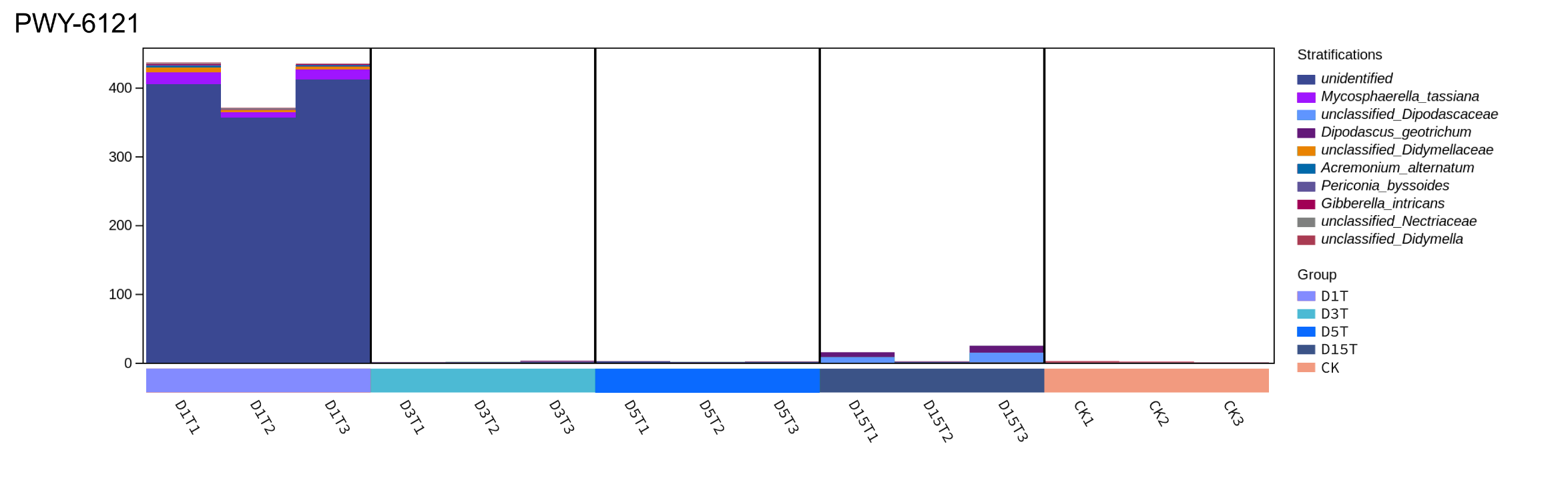

Supplement: Supplemental Information 6 — In this pathway, Alternaria was the dominant species in D1T, and only D1T was up-regulated compared with CK, while the other species did not exist in D3, D5, and D15. Therefore, the pathway was regulated by Alternaria. [file peerj-10-13286-s006.png]

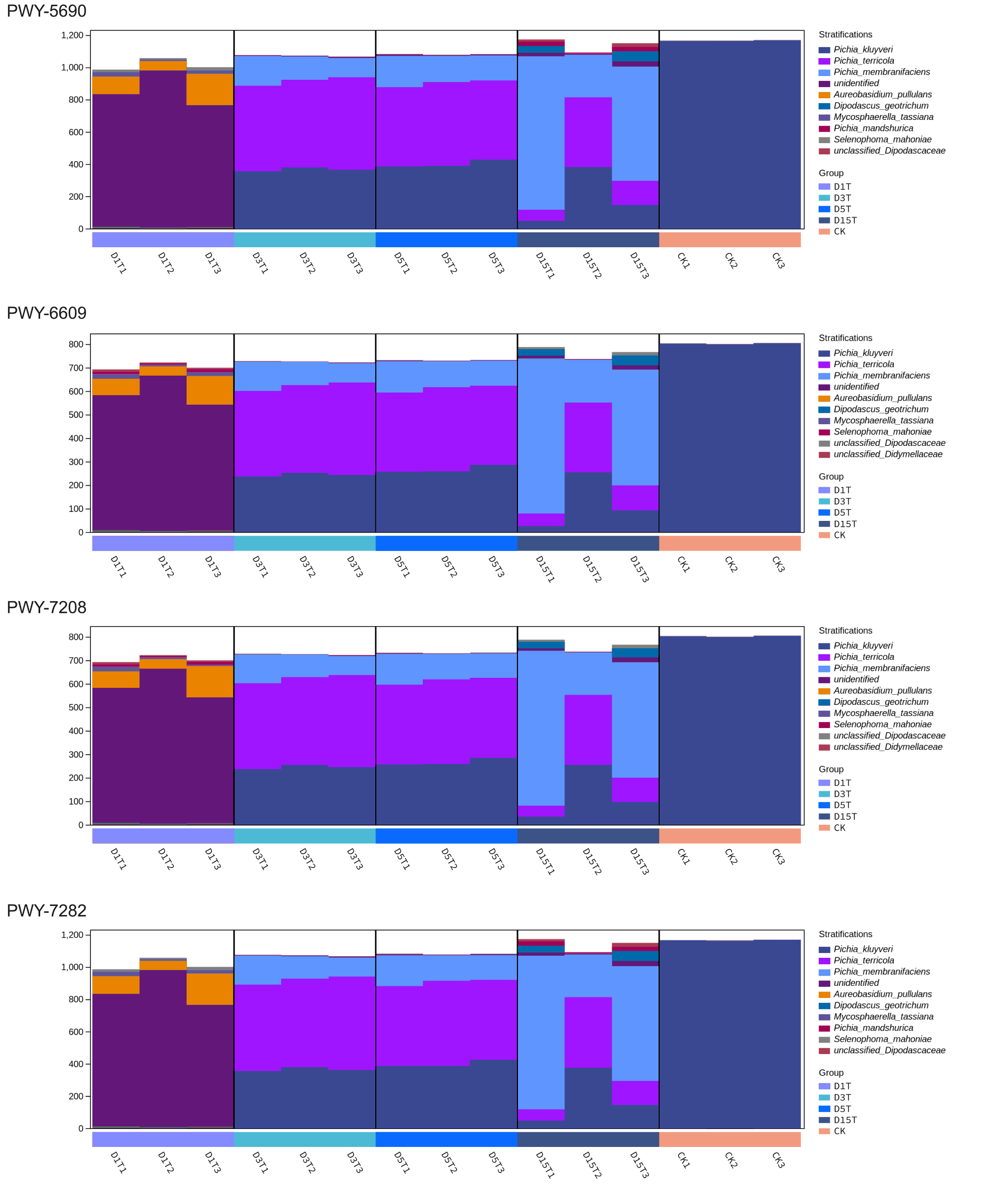

Supplement: Supplemental Information 7 — There was a small amount of unclassified Ascomycota in D1T and CK, but not in other samples. So these pathways were regulated by unclassified Ascomycota. [file peerj-10-13286-s007.png]
